# A Local Health Situation Room for COVID-19: Recommendations for Decision-Making From a Higher Education Institution in Mexico

**DOI:** 10.3389/fpubh.2021.735658

**Published:** 2021-10-25

**Authors:** Igor Martín Ramos Herrera, Diana Core Romero Lozano, Adolfo López Corona, José Francisco Muñoz Valle, Miguel Ernesto González Castañeda, Héctor Raúl Pérez Gómez, Marina de Jesús Kasten Monges, Moisés Ramos Solano, Norma Alicia Ruvalcaba Romero, Fabian Alejandro Pérez Ávila, Patricia Yokogawa Teraoka, Gabriela Macedo Ojeda

**Affiliations:** ^1^Department of Public Health, University of Guadalajara, Guadalajara, Mexico; ^2^Institute for Research in Biomedical Sciences (IICB), University of Guadalajara, Guadalajara, Mexico; ^3^Department of Molecular Biology, University of Guadalajara, Guadalajara, Mexico; ^4^Department of Geography and Territorial Ordering, University of Guadalajara, Guadalajara, Mexico; ^5^Department of Medical Clinics, University of Guadalajara, Guadalajara, Mexico; ^6^Department of Applied Psychology, University of Guadalajara, Guadalajara, Mexico; ^7^Department of Community Nursing, University of Guadalajara, Guadalajara, Mexico; ^8^Department of Social Sciences, University of Guadalajara, Guadalajara, Mexico

**Keywords:** COVID-19, public health, decision making, pandemics, epidemiology

## Abstract

**Introduction:** The Situation Room is a physical or virtual space where experts systematically analyze information to characterize a health situation, especially during emergencies. Decision-making processes are made toward solving health needs and promoting collaboration among institutions and social sectors. This paper presents the context and circumstances that led the University of Guadalajara (UdeG) to install a local health situation room (HSR) to address the COVID-19 pandemic at this institution based in the state of Jalisco, Mexico, a narrative is also made of its working processes and some of its results.

**Methods:** The design of this situation room for COVID-19 was based on the methodology established by the Pan American Health Organization (PAHO)/WHO. This local-type situation room was installed on February 12, 2020. The health problem was characterized, and strategic lines, objectives, and goals were established; the first analysis was derived from an action plan deployed at the UdeG. The strategic lines were situational diagnosis, preventive actions, and containment strategies.

**Results:** The situation room influenced the activities of the UdeG before the epidemic cases started in the state. One of the actions with the greatest impact was developing a mathematical model for predicting COVID-19 cases. Subsequently, new models have been developed according to the epidemiological evolution of the disease, helping manage the epidemic in the state. Another important result was the early closing of face-to-face university activities, reducing contagion risks and the mobility of more than 310,000 students, faculty, and administrative personnel throughout Jalisco.

**Conclusions:** A consequence of the closure was that the confinement generated by the pandemic was the change to virtual meetings from April 2020 to date; but at the same time, this working format was a strength, since it influenced the decision of the university board to change all the academic activities to virtual format before other educational, economic, and social activities in the state did. By April 2020, the situation room transcended its institutional boundaries and was invited to participate at the Jalisco State's Health Committee. Its recommendations have helped to maintain the state with one of Mexico's lowest COVID-19 incidence and mortality rates.

## Introduction

The Health Situation Room (HSR) is a term adopted from “war rooms”, which refers to closed physical spaces, which were housed in some secret place during wars where the military chiefs with knowledge about war strategies, international political conflicts, specialists in political communication, and key members of the government gather to analyze and decide the actions to take during war conflicts (1). It has been reported that there was a war room during World War II installed by Winston Churchill and that probably helped him to win together with the allied countries.

According to the WHO and the Pan American Health Organization (PAHO), epidemiology is a health science that is responsible for studying the distribution and the determinants of disease or health events and their application for the control of diseases and other health problems (2, 3). Epidemiology has successfully applied some terms and strategies that are used in wars to carry out its work, considering that the disease or event that affects populations is the enemy to be overcome. In this sense, the “war room” is a military strategy that has been transferred to the health field by the international organizations and some countries to attend to their health contingencies or emergencies, epidemic outbreaks, whether they be disturbing phenomena (natural, artificial disasters, etc.), administration of sanitary risks, epidemiological surveillance, cost-benefit analysis, and other events. By adapting these processes and activities, the “war rooms” were transformed into “HSR” (4) that are installed to carry out a situational health diagnosis (5) intended to respond to the health events through decision-making, based on timely and accurate information, and the experience of its members.

The situational health diagnosis later evolved to be called Health Situation Analysis (HSA). PAHO points out that HSA is the production of scientific evidence to support decisions on health issues (3). The generation of this evidence can occur through the collection of morbidity and mortality data, the analysis of these data, and the dissemination of the information obtained from the analysis. In many cases, the evidence comes from the results of research based on different studies used in health, such as population studies (3). But HSA has also been identified as management tools that serve to plan and prioritize health actions, optimizing economic, physical, and human resources, in addition to being an instrument that calls for intersectoral work. The goal of the HSA, therefore, is to contribute to the decision-making that allows solving the health needs of the population (6), with the intention of facilitating health management in a proactive, timely, and participatory manner, in addition to promoting collaboration with various actors and social sectors (7).

On the other side, the HSR is defined generically as the space where the HSA takes place. Even more, HSR is understood as “the physical and virtual space where health information is systematically analyzed by a work team to characterize the Health Situation of a population, especially during emergency situations. The information is presented and disseminated in various formats, such as tables, graphs, maps, technical documents, or strategic reports to make decisions based on the evidence; in this way, the HSR becomes an instrument for institutional management, political negotiation, the identification of needs, the mobilization of resources, and for the monitoring and evaluation of health interventions” (4). The main objectives of an HSR are to promote the use of epidemiology for health management, strengthening analytical capacities at different levels of the health system, respond to emergency situations based on epidemiology and HSA, make decisions based on evidence, and lay the foundations for the development of an epidemiological intelligence service with daily analysis of the health situation beyond the crisis (4).

The University of Guadalajara (UdeG) is the second-largest higher education institution in México with more than 310,000 students and is based in the state of Jalisco, Mexico. It is organized in 16 higher education centers (*campi*), more than 170 high schools, and one virtual education system, distributed all over the state. This conglomerate of *campi* and schools conform to what is called the University Network of Jalisco. The Health Science Center integrates more than 130 educational programs in the area of health, based at the *campus* located in the city of Guadalajara, capital of Jalisco.

The Council of the Health Sciences Center of UdeG approved, on February 14, 2020, the installation of an HSR to attend the COVID-19 pandemic under the Agreement Point 47/2020, which established that its creation was authorized for the purpose of “identifying, analyzing, proposing, notifying, coordinating, operationally and technically, the study, prevention, and intervention actions in different areas and levels of the university's community and the society, and regarding of the advance and development of the ongoing pandemic by SARS-CoV-2” (8). Therefore, the members of this HSR set the objectives of this work:

- The grounds and circumstances on which the UdeG installed a local-type HSR to attend the COVID-19 pandemic at the beginning of 2020 and the elements that were taken into account for its creation are first presented;
- The narrative of the initial analysis and decisions taken inside the room to lead the pandemic's attending efforts are then mentioned;
- Later, the account of the actions taken is made, along with its working processes and the main results obtained to date.
- Finally, the limitations of its results and the near future position of this higher education institutional health tool are described.

## Methods

The design of the UdeG's HSR for COVID-19 (UdeG-HSR) was based totally on the implementation methodology of Situation Rooms established by PAHO/WHO for the Region of the Americas (4). According to this methodology, there are four types of HSRs: local, county, state, and national. The one we are reporting here is a local-type HSR.

The objectives of an HSR should be (a) detecting and responding to communicable health problems on a timely, complete, and regular basis, (b) handling high-quality information, early detection, and prediction of epidemics, (c) providing an intervention plan during epidemics, and d) efficiently monitoring the planned intervention. These objectives are achieved through the implementation of four very precise actions: (1) training in epidemiology, (2) strengthening laboratories, (3) making communications more efficient in the health sector, and (4) giving special attention to health systems as a front line for health surveillance. According to these definitions, the operation of a situation room is based on three elements: the collection and processing of data, the analysis of the data, and the generation of the analysis products.

Therefore, installing this UdeG-HSR followed the next procedure, based on the aforementioned PAHO/WHO recommendations:

1. An initial request from the Provost of the Health Sciences Center was issued to the Department of Public Health, for the UdeG-HSR design and integration.
2. Invitations to the participants that would integrate at the UdeG-HSR.
3. Select the sources of information, the indicators that would be worked, and the databases that would be consulted.
4. Enable the physical space and the necessary resources to access valid and reliable data and information to carry out the HSA. This implied the use of statistical analysis systems, geographic information systems, trends, and inequity analysis systems.
5. The initial collection of the data available at the time of start-up and selection of the indicators that would be analyzed at the UdeG-HSR.
6. Encourage the analysis process so that the members of the HSR made the appropriate decisions based on evidence, with all the computer and communication resources during the sessions at the physical space, or remotely.
7. Constant and permanent collection of the data that would be generated and updated of the indicators subject to analysis by the participants.
8. Generate the elements to obtain an initial diagnosis, identify its determinants and risk factors, define priorities, carry out preventive actions, and identify potential needs for technical cooperation.
9. Generate specific prevention and effective communication actions for the entire community of the UdeG.

These recommendations were set at the forefront strategy of the UdeG-HSR with only minor adjustments. Nevertheless, the authorities and coordinators imprinted their particular operative functioning due to the conformation of the University Network and the local-type configuration of this HSR.

At the request of the provost of the Health Sciences Center, two meetings were held in early February 2020. During the first meeting, the pertinency for the creation of an HSR was identified, also the advantage of having that local-type HSR, according to the classification of PAHO/WHO (4), as a response tool for the University; another action at that first meeting was the profile definition of those who would participate at the room. In the second meeting, the invitation letters were delivered, and the roles of the invited faculty members, the characteristics of the meetings, and the physical working areas were defined. In this way, on February 12, 2020, the first formal meeting took place, aimed at installing it, and having the first working session at UdeG.

## Results

### Initial Analysis and Decision-Making

In January 2020, the Health Sciences Center and the whole University Network had already undertaken some prevention and sanitary actions for all the institutions, but it was not until the UdeG-HSR was formally installed that a catalog of data-based measures was communicated to all the institutional networks. To make this, the room was organized into two groups: the analysis group of the HSR (HSR-AG) and the HSA-extended group (HSA-e). The HSR-AG was integrated by a president (the President of UdeG), a general coordinator of The Provost of the Health Science center, an executive coordinator (the Chief of the Public Health Department), and six more specialists, while the HSA-e group was integrated by a technical coordinator, two PhD students, and 14 more members (9). After being informed of the existence of the UdeG-HSR, the Governor of Jalisco invited the president and the general coordinator of the HSR-AG, to participate at the State's Health Committee, to contribute to the analysis of the pandemic development at the state and dictate the actions to control it there. The comments and inputs made by these two representatives were the results of the HSR-AG and the HSA-e group's work.

It is worth mentioning that among the members of the HSA-e group, there were four epidemiologists, two molecular virus diagnosis researchers, three infectious diseases researchers, an expert in community nursing, one applied psychology, and one immunology researcher. During the meetings held over the first month, the pandemic problem at UdeG was elucidated and the strategic lines, objectives, and goals were established, which resulted in an Action and Execution Plan (10). This Plan was presented to the university general council on March 17, 2020, and distributed to all the University Network for its execution. The strategic lines presented on that Action and Execution Plan for the UdeG-HSR were the following:

1. Preventive actions (information for the university community). This strategic line allowed to estimate the intended coverage of the exposed population groups of the community, applying the most appropriate prevention or control measures, such as health protection, sanitation, and epidemiological surveillance.
2. Control strategies. Measures aimed at the prevention and protection of community health, necessary to adapt and create a culture of prevention.
3. University's Situational Diagnosis. Analyzing the new reality in the face of the COVID-19 pandemic, for decision-making, articulation, and directionality of plans, training programs, control, and monitoring.
4. Identification of the determinants and risk factors. Analysis of the indicators of determinants for the health status of the university community, recording vital activities and surveys for data administration.
5. Contact and support of suspected cases. Deployment of a surveillance program for those positive and suspected cases, by analyzing the data to provide care recommendations, and presentation of a proposal to strengthen prevention and containment measures.
6. Technical reports. Generation of analysis and results reports from the meetings, directed to the HSR-AG and the general council. Those reports contributed to the preparation of periodic messages to the university community.
7. Epidemic monitoring. Provide health information to all the University Network, monitoring the trends of the COVID-19 cases, through the analysis, interpretation, and contextualization of national, state, and local data.
8. Priority definition. The analysis allowed identifying priorities and offering interventions for decision-making aimed at evaluating the results and the impact it generated in the university community.
9. Evaluation. In terms of evaluation, the UdeG-HSR undertook a combination of quantitative and qualitative methodologies in order to promote compliance with the programs for the improvement of health actions, contributing to the orientation of strategies, fulfillment of the goals, and applying the planned methods and procedures with constant monitoring of the event.

These nine strategic lines guided the work of the UdeG-HSR, keeping its main objective in view and achieving the established goals. The main objective of the situation room was “to define the actions that will be carried out by the UdeG to attend the COVID-19 epidemic by SARS-CoV-2, both for the prevention of contagion and the diffusion of information toward the university community and the adequate handling of suspicious cases on the part of the dependencies of the Network.” Furthermore, four specific objectives were identified:

1. Disseminate the preventive messages of COVID-19 throughout the University Network.
2. Implement the infrastructure and resources to apply effective actions for the management of suspected COVID-19 cases.
3. Reduce the risk of contagion by COVID-19 at the university areas.
4. Continuously evaluate compliance with the objectives of the Action and Execution Plan.

Finally, the goals established at that time were the following:

1. An Action and Execution Plan for the prevention and management of suspected cases of COVID-19 was distributed to all the University Network on March 17, 2020.
2. All the University Network disseminating the information material on COVID-19 as of March 16, 2020.
3. All the University Network with access to a service area for suspected cases of COVID-19 as of March 20, 2020.
4. All the University Network with access to trained personnel to handle suspected cases of COVID-19 as of March 20, 2020.
5. All cases throughout the University Network with acute respiratory symptoms suggestive of COVID-19 were timely attended and adequately referred.

These elements were the starting work for three consecutive meetings in the generation of the Action and Execution Plan that served as the basis for the activities and recommendations that this HSR issued to the university authorities to face the COVID-19 pandemic in a timely manner and prevent, as established in the general objective, contagion among members of the university community, and the timely dissemination of quality information for their care. As of that date, the participants met every week 26 more times, physically until March 17, and virtually through videoconference, from March 24 to date.

### Working Procedures and Activities

The UdeG-HSR soon had direct involvement in the actions of UdeG. One of the elements with the highest impact was the implementation of the predictive model of COVID-19 cases, which was based on the incidence of cases and the national population parameters, to predict the possible number of cases that would occur on the following days/weeks. Section 3.4 will detail these models.

As of March 19, when the University closed its doors, the UdeG-HSR continued having weekly virtual meetings through a videoconference platform. This implied that the members became familiar with these platforms but did not restrict the wealth of contributions and observations that were generated to inform the university authorities about the pandemic evolution and support them in making decisions for the community.

As established in the strategic lines of the room, three technical reports were issued during 2020, which contained a series of recommendations for the management of the pandemic at the University. The characteristics of each report are:

- Technical Report no. 1. Issued on March 19, 2020, and recommended that university authorities recognize that COVID-19 was a real threat against which they should disseminate the appropriate information and be prepared to act; promote the active participation of the university community in actions for the prevention and containment of COVID-19 cases in accordance with the state, national, and international guidelines, and apply the guidelines of the Action and Execution Plan issued by this UdeG-HSR (not published and used for internal purposes only).
- Technical Report no. 2. Issued on April 7, 2020, and recommended that the university authorities continued with the academic activities of the 2020-A term in a virtual format until the end date of the courses scheduled by the General Coordination of School Control. Maintain administrative activities to a minimum in all *campi* of the University Network until the close of the 2020-A term and suspend the previously scheduled academic activities of the 2020 summer term, so that this period could be used for regularization activities of the 2020-A term, privileging non-contact modalities. Finally, integrate a Specialized University Committee to update the Institution's Educational Model that allowed to face this and other types of contingencies in a timely and flexible way, without affecting the performance of students, teachers, and administrative personnel (not published and used for internal purposes only).
- Technical Report no. 3. Issued on October 22, 2020, with the annual activity report of the UdeG-HSR, in which all the work carried out, the projects that derived from it and the communication and dissemination work of scientific and preventive information for the university community was reported (not published and used for internal purposes only).

Weekly reports were carried out in which data were collected by selecting the population indicators for epidemic surveillance based on the most appropriate sources of information, through the constant updating of databases and from the information that came from the different agencies, such as the Ministry of Health (both federal and state levels) official technical communications. Subsequently, an analysis was carried out to support the management of the monitoring and evaluation of the scenario. Those reports were created based on the preparation of information sheets, in addition to carrying out the corresponding preparation of the presentations for each session. Those presentations included fundamental sections where the agenda of the day was presented.

In addition, these activities were made: a series of TV, journals, and radio interviews were given by the members of the UdeG-HSR; a dissemination plan for the university community where active participation in the mass-media was carried out; on a preventive basis, information campaigns were designed and published by the Social Communication Office with prevention measures, official announcements, and news, for example, “take care of COVID-19” and “protect yourself”, among others. The General Provost handed nine reports and memos related to the work of the UdeG-HSR, 12 press conferences were organized by the UdeG Media office, 68 press releases highlighted by the UdeG press office, and about 200 “Coronavirus, The Pandemic” TV programs cast by channel 44 (UdeG official TV channel). The official communication website was created to publish all the information generated; in addition, Google Trends reported 873 direct searches with the word “Test COVID UdeG”, 384 direct searches with the word “COVID UdeG”, 32,800 results associated Google with a “UdeG Situation Room”, and 125,000 results associated with the “UdeG Situation Room”, with 350 interviews in different media. All this activity occurred to sensitize the university community to maintain prevention measures and actions against the COVID-19 pandemic.

### Most Remarkable Results

One of the actions that have had the highest response on our university community was advising the different *campi* and high schools on how to proceed to prevent the disease. This information, together with the Action Plan designed by this HSR, was implemented through the operative teams. These teams were called Operative Rooms or Auxiliary Commissions at the different centers of the University Network depending on their physical and community size.

During the execution of the Action Plan, a large number of direct and indirect results were obtained, some of the direct results are the abovementioned predictive models that estimate the magnitude of the epidemic in the state of Jalisco and the University Network, with monthly updates of the disease; the Geospatial Analysis of Jalisco and the central-western region of Mexico, through 24 presentations and 11 special reports published on the official website of the pandemic, focusing on the analysis of suspected and confirmed cases, and incidence and mortality rates at different levels of disaggregation; permanently monitoring the epidemiological panorama at international, national and state levels, and the preparation of a weekly synthesis and epidemiological report, was presented at every meeting of the HSA-e group. Another activity was the training of the University Network teams, based on the Operative Model of the University Network creating a MOOC-type course-workshop which allowed to face from the beginning any eventuality and handling of suspected cases at the university network, this activity could not be deployed without the collaboration of the Medical Unit of UdeG. The Integral Psychological Care for Well-being Clinic (CAPIB) was created offering online psychological counseling for students, free of charge, with 24/7 service; the Strategy for the Diffusion and Dissemination of Science program was created to develop the information campaigns aimed at the university's and state's community; also, the HSR-AG designed the University Epidemiological Surveillance Project, establishing the bases and guidelines for epidemiological surveillance at the return to personal classes at the university facilities; other research projects that are in process from the departments of Molecular Biology, Public Health, and Applied Psychology, these projects are detailed below.

Indirect results obtained by the work of the UDG-HSR are the following: a Call-Center was established at the UdeG that, as a support for the Ministry of Health of the Government of Jalisco, which goal was to help the population to identify the risk of having the disease and, when appropriate, schedule them for the RT-PCR, antigen or antibody tests at the COVID-19 diagnostic laboratories; the COVID-19 Diagnostic Laboratories was organized in a Diagnostic System, which came into operation on April 16, 2020 and included 10 diagnostic laboratories at the University Network where they run the tests as a Drive-Thru service; this system also includes the Rapid Test Center for antigens and antibodies detection. Another indirect result is the participation at the Radar Jalisco strategy, coordinated by the Ministry of Health of Jalisco, where the laboratories of UdeG and the Civil Hospital of Guadalajara collaborated in the application of RT-PCR tests and reporting the results. Another important result is the online system that evaluates the risk of contagion, designed by a group of researchers at the Engineering Sciences Center, this system is offered on a website that is linked to the Ministries of Health (state and federal) where individuals can evaluate the infection risk on recent days and make specific recommendations according to the result obtained. The project proposal “Am I a COVID-19 case?” with the collaboration of a group of physicians working at the Civil Hospital of Guadalajara that addresses the crisis or lack of information in the population about the symptoms and the procedures to follow in case of being virus carriers or having health complications.

### The Predictive Models and Other Research Projects

To estimate the magnitude of the pandemic, as described before, the HSR-AG developed several predictive models. The first model was presented on February 28, 2020, as a written report at the HSA-e group meeting, this model was based on epidemiological data from the John Hopkins University & Medicine, Coronavirus Resource Center (11), and the Mexican Ministry of Health data resource site (12) monitoring systems. This model located the day on which there would be a turning point for the increase in cases, from which the HSR-AG raised a recommendation to close the University on March 19, 2020. On March 30, the HSR-AG invited a group of mathematical modeling experts from our University and data science associations to create a new model. Its objective was to estimate the increase in infections in the following days and the level of citizen participation in the prevention measures implemented by the state, based on the SEIR model (which considers the susceptible, exposed, infected, and recovered population). Based on this analysis, a 40% citizen participation in prevention measures was estimated. The model recommended implementing measures to achieve 60% participation, estimated to avoid exceeding the state's hospital capacity. Consequently, the state authorities considered this recommendation and strengthened the supervision of prevention measures and communication of risks to the population.

Later this year, the REPLICA model was created in collaboration with researchers from the University of California and the University of Georgia (13). The REPLICA model contributed to decision-making for the economic reactivation of the state in a staggered manner and according to the geographical distribution of economic activities in the state. The model showed that closing schools and using face-masks would maintain contagion below the line of saturation of health services. Authorities of Jalisco State implemented the measures and kept Jalisco with one of the three lowest incidence rates in Mexico last year that otherwise will put in trouble the health system. The detailed technical reports on the construction of these models and the recommendations issued from them are published on the website of the UdeG (14) and were disseminated through press conferences.

These reports had a high influence in determining the academic activities at the university, advising to continue online classes, and keeping students and teachers at home, to avoid or reduce the risk of contagion. These reports served also as input to the Health Committee of Jalisco for the decisions and actions that took place at the state level. The main limitation of these models was the imprecision due to the different factors involved. However, they gave an overview of trends, which contributed to more informed decisions.

Scientific progress during the health contingency was also essential to know the behavior and development of the pandemic. For this reason, some members of the HSR-AG along with other UdeG researchers developed a group of scientific projects that contributed to this aim, and their results have already been published or still in press. Some of these projects are:

- Vitamin D and COVID-19: A review on immunomodulatory effects of vitamin D in the prevention of severe COVID-19 (15);
- Vitamin D Levels in COVID-19 Outpatients from Western Mexico: Clinical Correlation and Effect of Its Supplementation (16);
- Association of Food Intake Quality with Vitamin D in SARS-CoV-2 Positive Patients from Mexico: A Cross-Sectional Study (17).
- Factors related to COVID-19: COVID-19 Screening by Anti-SARS-CoV-2 Antibody Seropositivity: Clinical and Epidemiological Characteristics, Comorbidities, and Food Intake Quality (18).
- COVID-19 diagnosis: RT-qPCR Assays for Rapid Detection of the N501Y, 69-70del, K417N, and E484K SARS-CoV-2 Mutations: A Screening Strategy to Identify Variants With Clinical Impact (19).
- Effect of vaccination against COVID-19: Neutralizing Antibodies Titers and Side Effects in Response to BNT162b2 Vaccine in Healthcare Workers with and without Prior SARS-CoV-2 Infection (20).

## Discussion

The main results of the UdeG-HSR implementation were (1) creating an HSR based on the PAHO/WHO model to respond to health problems as other Latin American countries have done; (2) organize its members in two groups, the HSR-AG and the HSA-e groups; (3) the support to the institutional and state's governmental levels; and (4) effective communication of the results to the university network and the community of Jalisco.

### UdeG-HSR Compared to Other Latin American HSRs

The PAHO/WHO model for HSRs defines a specific set of principles. According to the level of application, they can be national, state, county, or local type. In comparing the functioning and results of UdeG-HSR with other Latin American experiences we found that in Argentina, Brazil, Costa Rica, Venezuela, and Mexico, there have been similar efforts, following the same PAO/WHO model but sometimes with different objectives (not everyone attending COVID-19). In Argentina to start, the federal government installed a state-type HSR on each one of its departments, aimed to analyze the situations of departments and to generate actions according to each department's characteristics (21), but following the same steps as the UdeG-HSR. From that point, a National Program of Healthy Cities, Municipalities, and Communities was created to identify health problems in their departments, prioritize them, and define the plans and programs that would be implemented, all from the perspective of healthy municipalities and communities (6, 21).

A different experience took place in Brazil in 2010, when they promoted the use of data to offer dynamic diagnoses and improve the health of the population through a National-type HSR, thus making it possible to prepare plans that were compatible with the identified needs, to promote the improvement of the health care system which results allowed the application of new public health policies (22). In Costa Rica, they used to have expert meetings whose main purpose was the discussion toward consensus for the standardization and dissemination of guidelines in health services for daily practice. A methodological proposal was developed which included the development of strategic lines, as UdeG-HSR did, that resulted in the description of the historical, political, socioeconomic, and demographic context of the population, in addition to the analysis of the quality monitoring statistics. The data on the life and wellbeing of the population served for the identification of priorities proposing health interventions to be able to evaluate the impact of public policies, programs, and health services promoting social participation (23).

The National-type HSR created in Venezuela in 2018 intended to have a better interpretation of the health and disease processes. All the administrative processes could be understood in a better way since the planning prior to the intervention at the cities and departments. Analyzing data in a more accurate way for the main causes of morbidity and mortality, health promotion, and approach to public policies to meet local, national, and regional indicators (24).

Regarding the current pandemic, again in Argentina a COVID-19 National-type HSR was created, where an epidemiological analysis of the disease situation was carried out at the international, regional, and national levels (25). All the cases were studied and reports were issued for the federal government and recommendations were communicated to the community. Epidemiological and genomic surveillance reports were also made every month to provide updates on the current situation of the disease. They have been reporting since March 2020, like UdeG-HSR. The government of Costa Rica created a National HSR on July 28, 2020, in response to the COVID-19 pandemic. They hold a permanent meeting, as the HSA-e group has made, where the Ministry of Health and the Social Security were converted to emergency care areas. Permanent evaluations of the epidemiological indicators are carried out in quantitative analysis (23).

Finally, in Mexico, the response to the disease involved timely and immediate actions by various states of the Republic, operating manuals for the epidemiological and sanitary intelligence units were published by the Mexican Ministry of Health, where the implementation of Crisis Rooms and Intelligence Rooms constituting another epidemiological tool for decision-making in Public Health based on Evidence (26), however, the federal government did not call them HSRs. Such is the case of Oaxaca, a southern state of Mexico, where a crisis room was implemented as part of a COVID-19 Health Operational Command (27), whose purpose was intersectoral integration, generating a link with the “Emergent Plan of Comprehensive Approach for the Prevention and Control of Respiratory Infection by COVID-19 in the state of Oaxaca”, where joint agreements were reached establishing the objectives and strategies to carry out the corresponding activities.

As can be seen, these Latin American countries have implemented situation rooms at different levels, they all used the same PAHO/WHO model as the UdeG-HSR. The grounds and processes remain similar along with these countries, though some operating activities and implementation have been different. In the end, the results are very similar: a group of experts addressing a health situation based on quality data, adequate analysis, and a strong methodology and support from the authorities.

### Organization and Limitations

The UdeG-HSR has worked under the situation room model generated by PAHO/WHO for more than 1 year. Though it followed the recommendations for its organization and operation, this HSR was organized into two groups: the HSR-AG and the HSA-e groups. This arrangement was considered to widen its scope at the university network because the first group was focused on the analysis of the evolution and impact of the pandemic, and the second was to recommend the strategies and operative activities at the institution.

However, in light of the different contexts where HSR have been implemented in other Latin American countries (28), we identify that this room would be limited in its actions because of the Local-Institutional type, that is, it was not installed by a county, state or federal health ministry; therefore, the impacts may not reflect the actions as widely and adequately as we would expect. Another situation is that not all the faculty or experts who make decisions at the University Network participate in the room, and this can constrain the decisions taken or the actions derived from it. The impact, as mentioned before, maybe reduced for these and other reasons, but it can only be measured when the corresponding evaluation will be carried out at the end of 2021 as planned.

The University Network is ready to receive students and faculty, the return to the university has been delayed until October 15, 2021, however, the UdeG-HSR and the authorities have declared that the control of the epidemic lies in joint responsibility with the university community. A return-to-classes plan has already been prepared to keep everyone safe when the conditions are set properly in Jalisco and Mexico. At the beginning of 2021, the Provost of the Health Sciences Center authorized the continuity of the situation room for this year, with the agreement of the University's President. Both groups will continue working with minor changes and additions in their integrating members and their functions.

### Government Support and Indirect Results

Most of the reported projects are concluded, but there are some other research projects and publications in planning or development, they are focused on the epidemiologic, molecular biology, public health, and psychology characteristics of the disease. The internal activities will also continue and the two groups will maintain their collaboration with the state's Health Committee and with other Universities or Institutions, to support a positive Public Health impact on the population, containing or controlling the pandemic. The indirect results of the UdeG-HSR were mainly over the activities of the state, thanks to the openness of the ministry of health and the governor of Jalisco. It is clear that an institutional local-type HSR cannot limit its work for in-house benefit only; therefore, the results and impact will go beyond its boundaries and surely will affect the near society.

### Communication and Impact on the Community

In sum, the creation of the local-type UdeG-HSR had a positive impact as it provided data-based decisions and communication resources to issue the necessary recommendations and strategies as a response to the COVID-19 pandemic, whose main focus was monitoring and responding to a health problem with public health measures, identifying the risk factors and predicting its spread rhythm and time.

One of the most evident impacts of the UdeG-HSR is the incidence and mortality rates at the state of Jalisco, reported among the lowest in the country by the time this article was written. For instance, in January 2021, the federal government reported that there were 53,810 confirmed cases and 6,254 deaths in Jalisco due to COVID-19, the fifth-highest (out of 32) in Mexico in both indicators (29). In spite of these absolute numbers, the preventive measures implemented at the state took the incidence rate to 31.9 cases per 100,000 inhabitants, and the death rate was 74.3 deaths per 100,000 inhabitants, which located Jalisco at the 13th and 28th positions in Mexico, respectively (29, 30), both of them below average, and among the middle and lowest in the country.

## Conclusions

This study shows the actions taken by the UdeG-HSR in face of the COVID-19 pandemic. The conclusions are:

1. This HSR was created as a local-type room, therefore, its scope was limited to the institution's boundaries. However, due to the relationship with the state government, its actions and recommendations went beyond it.
2. The timely installation and work of the UdeG-HSR helped the state of Jalisco to maintain one of the lowest incidence and mortality rates in the country, in part because the institution maintained virtual activities since last year avoiding the mobilization of more than 310,000 students, faculty, and administrative personnel.
3. There were direct and indirect products of the situation room, one of the most important was the mathematic models to analyze the pandemic evolution, which has evolved.
4. Communicating campaigns and programs were effective to target the university community; the response was immediate and kept everyone at home.
5. The evaluation of impact and effectiveness has not been done, yet it has been scheduled for late 2021. This evaluation requires a validated methodology and analysis criteria that are at a preparation phase, as mandated at the Action and Execution plan.

## Data Availability Statement

The original contributions presented in the study are included in the article/supplementary materials, further inquiries can be directed to the corresponding author/s.

## Author Contributions

JM and GM contributed to the conception of the UdeG-HSR. JM, GM, IR, DR, and AL contributed to establishing its fundament. JM is the general coordinator of the UdeG-HSR, represents it in the health group of the State Government, and has managed its relationship with different sectors. GM is the executive coordinator of the ISS analysis group and has represented it in the state government health group. IR was the technical coordinator for the expanded UdeG-HSR group. IR, DR, and AL wrote the first draft of the manuscript. All the authors contributed to critically reviewing it for important intellectual content and approved the submitted version.

## Conflict of Interest

The authors declare that the research was conducted in the absence of any commercial or financial relationships that could be construed as a potential conflict of interest. The handling editor declared a past collaboration with one of the authors IR.

## Publisher's Note

All claims expressed in this article are solely those of the authors and do not necessarily represent those of their affiliated organizations, or those of the publisher, the editors and the reviewers. Any product that may be evaluated in this article, or claim that may be made by its manufacturer, is not guaranteed or endorsed by the publisher.
